# Examining Atherosclerosis Patterns in the Circle of Willis: A Case Study of Duplicated Anterior Communicating Artery

**DOI:** 10.7759/cureus.53321

**Published:** 2024-01-31

**Authors:** Tara Tritsch, Mohammadali M Shoja, Andrew Schleffer, R. Shane Tubbs

**Affiliations:** 1 Department of Medical Education, Nova Southeastern University, Dr. Kiran C. Patel College of Allopathic Medicine, Fort Lauderdale, USA; 2 Department of Anatomical Sciences, St. George's University, St. George's, GRD; 3 Department of Neurosurgery and Structural and Cellular Biology, Tulane University School of Medicine, New Orleans, USA

**Keywords:** variations, cerebral, atherosclerosis, artery, anatomy

## Abstract

The anterior communicating artery (ACoA) plays a pivotal role in maintaining cerebral hemodynamics, as its diameter is a major determinant of blood collateralization through the circle of Willis following internal carotid artery occlusion. While variations of this artery are not uncommon, data on their clinicopathologic relevance are limited. In this report, we present our observation from a fresh cadaver of a male individual who had succumbed to cardiac causes. The circle of Willis displayed a duplicated ACoA with atherosclerosis that predominantly affected the posterior horn while sparing the anterior horn. The anterior horn was characterized by its shorter length and larger diameter compared to the posterior horn. The paper focuses on elucidating the microsurgical anatomy of this particular ACoA variant and exploring potential mechanisms that may underlie the pattern of atherosclerotic distribution within the circle of Willis. Based on this report, while further evidence is needed for confirmation, it is plausible that the existence of a duplicated ACoA may offer a protective mechanism, ensuring uninterrupted collateral circulation in the event of a blockage in one of the horns. Further analysis of the ACoA and its pattern of involvement in intracranial atherosclerosis is warranted, as the atherosclerotic patterns in this region hold clinical and pathological significance.

## Introduction

The anterior communicating artery (ACoA) constitutes the foremost segment of the circle of Willis, playing a pivotal role in cerebral hemodynamics as its diameter significantly influences collateral blood flow in the presence of an occluded and atherosclerotic internal carotid artery [1]. Although variations in the ACoA are not uncommon, a comprehensive understanding of their clinicopathologic significance remains elusive [2-6]. Stopford identified seven distinct ACoA variations, including absence, duplication, triplication, quadrupled forms, and even a plexiform or network-like ACoA [2]. Kleiss expanded on these variants by considering their relationships with the anterior cerebral artery (ACA) and the overall configuration of the circle of Willis [3]. Among these variations, duplication of the ACoA is the most prevalent [2,4]. Stopford recognized three types of duplication: complete duplication, V-shaped duplication, and Y-shaped duplication [2]. In some cases, an accessory (or third) ACA may arise from either the anterior or posterior horn of a duplicated ACoA [2,3]. The segmental duplication or fenestration at the ACA-ACoA level is believed to result from incomplete fusion of precursor arteries around day 35 post-conception [5].

The incidence of duplicated ACoA varies across different series, ranging from 7% to 34% [1,4]. The frequent occurrence of a duplicated ACoA in conjunction with aneurysms has spurred speculation that this variant of the circle of Willis may be associated with structural abnormalities in the arterial tunica media [5-10]. We present a case of duplicated ACoA that exhibited significant atherosclerosis, primarily affecting the posterior horn while sparing the anterior horn. Cerebral atherosclerosis was graded macroscopically according to Baker’s scoring [11]. This includes grades I-IV, ranging from localized arterial opacity without luminal narrowing (grade I) to complete vessel circumference involvement with marked luminal narrowing (grade IV). The discussion focuses on elucidating the microsurgical anatomy of this particular ACoA variant and exploring potential mechanisms that may underlie the pattern of atherosclerotic distribution in the circle of Willis.

## Case presentation

**Figure 1:**
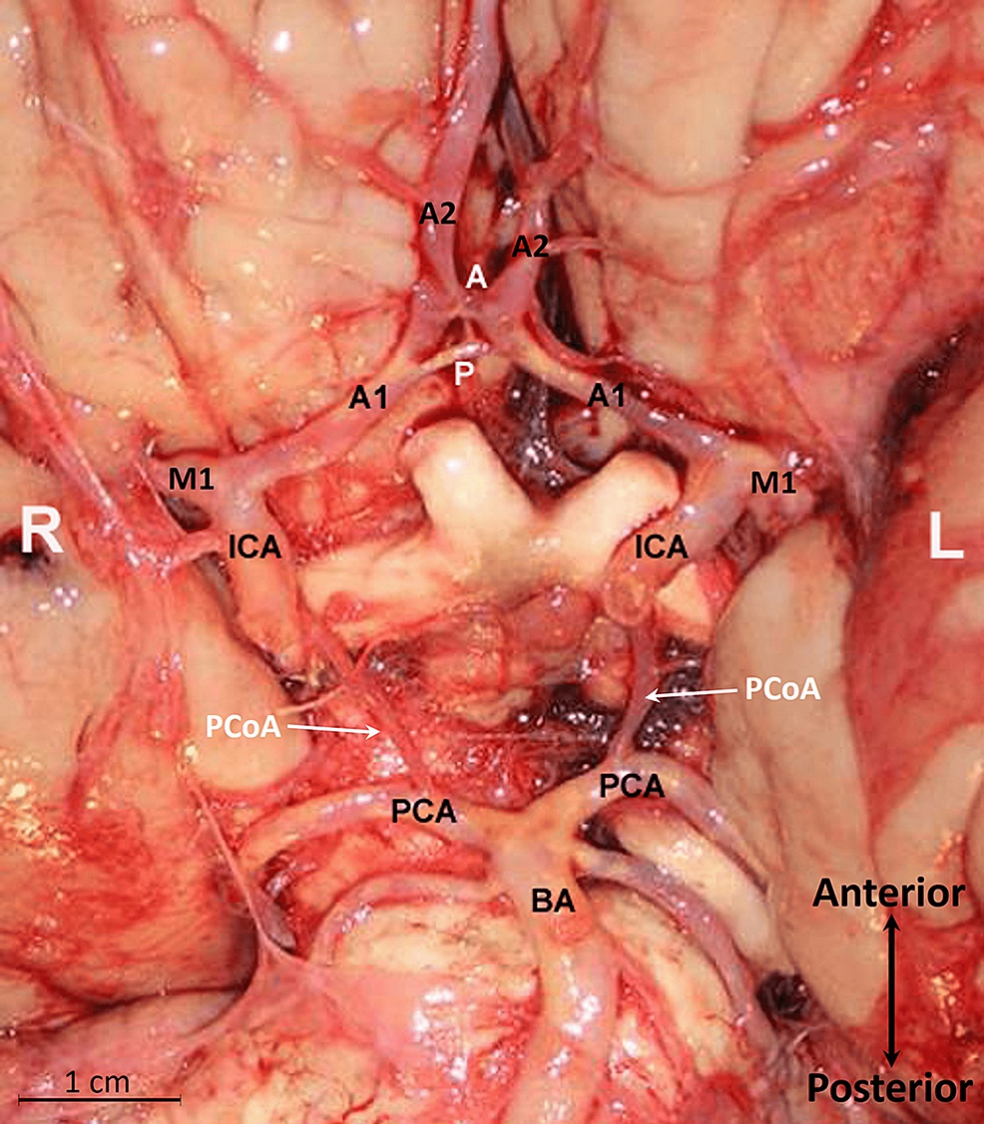
The base of the brain and the circle of Willis The internal carotid artery (ICA), posterior cerebral artery (PCA), basilar artery (BA), and A1 or first segment of the anterior cerebral artery (ACA) are atherosclerotic (yellowish hue). The anterior communicating artery (ACoA) is duplicated, featuring anterior (A) and posterior (P) horns, with a small vertical bridging artery connecting the two horns. Notably, the posterior horn exhibits severe atherosclerosis. The right (R) and left (L) sides are indicated. A2, the second segment of the ACA; M1, the first segment of the middle cerebral artery; and the posterior communicating artery (PCoA).

The dissection was performed on a fresh cadaver from a 63-year-old Caucasian male who had succumbed to cardiac causes. To access the intracranial cavity, the calvaria were removed, and the brain was extracted following a cut along the attached border of the tentorium cerebelli and transection of the spinal cord at the level of the foramen magnum. A duplicated ACoA was noted, without other significant arterial variations in the circle of Willis (Figure 1). The ACoA consisted of two horns: an anterior horn and a posterior horn. The anterior horn measured 2 mm in length and had an external diameter of 2 mm, while the posterior horn measured 4 mm in length with a 1 mm external diameter. A fine arterial communication (or bridging artery, 0.8 mm in diameter) connected the anterior and posterior horns. The origin of the anterior horn of the ACoA was perpendicular to the plane of the ACA, whereas the posterior horn had an acute angle of origin. Two long penetrating branches originated from the superior surface of the anterior horn, coursing posteriorly and superiorly as the anteromedial group of perforating arteries to supply the lamina terminalis and suprachiasmatic region. The posterior horn had no branches and followed an oblique course. The fine-bridging artery, primarily located to the left of the midline, measured 1 mm in length. On gross inspection, atherosclerosis was evident in the internal carotid arteries, posterior cerebral and basilar arteries, and the A1 segment of the ACA. Notably, the entire posterior horn of the ACoA, including the bridging artery, exhibited severe atherosclerosis (grade IV), while the anterior horn showed minimal atherosclerotic changes (grade I). No other gross intracranial arterial variations were identified in this brain specimen.

## Discussion

Examining the atherosclerotic patterns within the circle of Willis and their correlation with arterial anatomy holds significance from a pathophysiological standpoint. In the present case, we observed that, under the influence of the same systemic hemodynamics, the posterior horn of ACoA exhibited severe atherosclerosis, while the anterior horn showed minimal atherosclerotic effects. This discrepancy warrants further explanation. According to Laplace's law, the tension in a vessel's wall is directly proportional to its radius [11]. Therefore, larger arteries are generally more prone to atherosclerosis [11-13]. Resch et al. and Reed et al. reported that among cerebral vessels, the communicating arteries tend to have a lower propensity for atherosclerosis due to their smaller caliber [11,14]. In this context, one might expect that the anterior horn of the ACoA in the present case, with its diameter twice that of the posterior horn, would be subjected to higher tension and, consequently, atherosclerosis. The observations in this case raise a compelling point: arterial size may not exclusively dictate atherosclerosis susceptibility within the confines of the circle of Willis. Local geometric parameters are recognized as among the significant risk factors for atherosclerosis [15]. It is plausible that the acute angle of branching of the posterior horn of the ACoA, in conjunction with its proximity to larger arteries (e.g., internal carotid and middle cerebral arteries), may have exposed this vessel to increased hemodynamic turbulence.

The diameter of the ACoA can vary significantly, ranging from 0.1 to 4.9 mm [1,16]. Tulleken defined a normal ACoA as having a diameter of 2.0-2.5 mm, a criterion met in only 20% of cases [17]. ACoA with an external diameter of 1.0 mm or less is sometimes referred to as "small" or "string-like" ACoA [1]. The reported incidence of this variant ranges from 3% to 37% [18-19]. Studies have shown that the mean diameter of a duplicated ACoA is typically smaller than that of a single artery [16-17,20]. Additionally, Cassot et al. have suggested that a duplicated ACoA exhibits higher arterial resistance [1]. While a duplicated ACoA is frequently documented in the literature, its functional significance and pathological relevance remain largely unexplored.

Some reports have linked a duplicated ACoA to the presence of aneurysms within its anterior horn [6-7]. However, the association between a duplicated ACoA and atherosclerosis, as well as the specific patterns of such atherosclerosis, has yet to be described. While there is a paucity of literature regarding the connection between anatomical variations in the anterior part of the circle of Willis and ischemic brain lesions, there is evidence to suggest that variations in the posterior circulation may be associated with ischemic events [21-22]. In a study of patients with suspected cerebrovascular accidents, the presence of ipsilateral posterior communicating artery hypoplasia or aplasia was associated with a higher frequency of thalamic infarction [21]. Another study revealed a significant association between the presence of the fetal posterior cerebral artery and covert vascular brain injuries within the general population [22]. Therefore, future studies should also address the relationship between anatomical variations in the anterior portion of the circle of Willis, atherosclerosis, and ischemic brain lesions.

Atherosclerosis of the circle of Willis

The atherosclerotic patterns involving the circle of Willis have been explored in previous studies [23-27]. It is established that the larger intracranial arteries, such as the internal carotid, middle cerebral, basilar, and vertebral arteries, frequently exhibit more extensive atherosclerotic damage compared to the smaller segments of the circle of Willis [20,23-26]. In addition to the hypothesis related to Laplace's law mentioned earlier [11], another proposed explanation for this observation involves a decrease in blood pressure during the transition from larger to smaller caliber intracranial arteries. This pressure drop could potentially act as an intrinsic mechanism to safeguard the smaller arteries within the circle of Willis from developing atherosclerotic plaques [25]. Fewer studies have examined or included the ACoA when analyzing the distribution of atherosclerotic plaques within the circle of Willis. Denswil et al. discovered that among 33 samples, only 10% of the examined ACoA arteries exhibited signs of advanced atherosclerosis [25]. Wijesinghe et al. reported evidence of no or only mild atherosclerosis in 80% of the ACoA arteries examined, moderate atherosclerosis in 17.1%, and severe atherosclerosis in 2.8% [26]. In a study by Llopis et al., which examined a total of 261 atherosclerotic plaques, only two were identified in the ACoA [27].

## Conclusions

In a nutshell, comprehending the clinicopathological implications of the circle of Willis anatomical variations may offer insights into potential disease mechanisms. A duplicated ACoA may provide a protective effect, ensuring ongoing collateral circulation in the event of a blockage in one horn. Since ACoA plays a key role in blood collateralization through the circle of Willis, the anatomical correlates of its atherosclerosis, such as diameter, length, and anatomical variations, need to be explored in future studies. This case study presents one such observation and highlights that factors beyond arterial diameter may play a role in determining the presence of atherosclerosis within smaller segments of the circle of Willis.
